# Sensory eye balance in surgically corrected intermittent exotropes with normal stereopsis

**DOI:** 10.1038/srep13075

**Published:** 2015-08-19

**Authors:** Lixia Feng, Jiawei Zhou, Li Chen, Robert F. Hess

**Affiliations:** 1 Department of Ophthalmology, First Affiliated Hospital, Anhui Medical University, Hefei, Anhui, PR China; 2 McGill Vision Research, Dept. Ophthalmology, McGill University, Montreal, PQ, Canada

## Abstract

Surgery to align a deviated or strabismic eye is often done for both functional as well as cosmetic reasons. Although amblyopia is often an impediment to regaining full binocularity in strabismics in general, intermittent exotropes, because their deviation is intermittent, have no amblyopia and some degree of stereopsis. Binocular function, including a balanced ocular dominance, could be expected to be normal after surgical correction if normal levels of stereopsis and visual acuity are postsurgically achieved. Here we used a binocular phase combination paradigm to quantitatively assess the ocular dominance in a group of surgically corrected intermittent exotropes who have normal stereo and visual acuity as defined clinically. Interestingly, we found significant interocular imbalance (balance point < 0.9) in most of the surgically treated patients (8 out 10) but in none of the controls. We conclude that the two eyes may still have a residual sensory imbalance in surgically corrected strabismus even if stereopsis is within normal limits. Our study opens the possibility that a further treatment aimed at re-balancing the ocular dominance might be necessary in surgically treated intermittent exotropia to provide more efficient binocular processing in the long term.

Strabismus, a condition in which the eyes are not properly aligned with each other, affects approximately 1–4% of the population across the world^1^,^2^,^3^,^4^. Strabismus may be associated with amblyopia if it occurs early in visual development^5^. Several therapies have been proposed for treating ocular misalignment of strabismus including refractive correction^6^, eye exercises^7^, botulinum toxin therapy^8^ and surgical alignment^9^. Of these, surgery is a commonly used treatment especially for patients with a large angle of misalignment^10^. Previous studies have shown that in about 30%–75% of cases binocularity and stereopsis are improved following successful surgical alignment^11^,^12^,^13^,^14^,^15^,^16^,^17^,^18^,^19^. This is reasonable as many nonamblyopic strabismics may have intermittent fusion with proper ocular alignment during the early stage of life, which in turn enabled development of binocularity and stereopsis before the onset of the strabismus. This has been demonstrated frequently in one category of strabismus: intermittent exotropia, which occurs in about 1% of children by the age of seven years in US^20^. These patients could have stereovision both preoperatively and/or postoperatively when the eyes are aligned^15^,^21^,^22^,^23^. Since stereopsis is often the gold standard for binocular vision^24^, we want to ask a very specific question about whether the two eyes contribute equally to binocular perception (i.e., the ocular dominance) in surgically corrected intermittent exotropes who have normal postoperative stereo vision (i.e., less than 100 arc sec in the clinical stereo tests using Random-dot stereograms and Frisby). Intermittent exotropes would be expected to have the best chance of achieving balanced ocular dominance post surgery, as there are no impediments in their early visual development. In fact, there is evidence to show that the sensory outcome of intermittent exotropia is better than that of constant exotropia^25^. However, intermittent exotropes could exhibit residual binocular abnormalities since at least for part of the time they were strabismic before surgery and during this time presumably information from the fixing eye suppressed information from the strabismic eye. The assessment of ocular dominance for this special clinical population, i.e., surgically corrected strabismus with normal clinical visual acuity and stereopsis, is important to establish whether they are indeed “binocularly normal” in all respects.

To answer this question, we quantitatively assessed the sensory eye dominance in a group of 10 teenagers/adults who had intermittent exotropia with no acuity loss prior to the surgery and who were, postsurgically, orthotropic or having a horizontal heterotropia of 10 prism diopters or less with normal stereopsis (defined clinically as less than 100 arc sec in the clinical stereo tests using Random-dot stereograms and Frisby). We used a binocular phase combination paradigm^26^,^27^ to assess the interocular contrast difference that was needed to result in a balanced binocular combination. Previous studies have used this paradigm to show that the two eyes of binocularly normal individuals are equally effective when the visual inputs have equal contrast^26^,^28^,^29^,^30^, indicating a sensory eye balance. However, for conditions when one eye is less effective than the other eye due to unilateral amblyopia^27^,^31^,^32^,^33^ or unilateral luminance reduction^30^, additional contrast of the visual input is needed for this eye to achieve a balance with that of the other eye. We found that, interestingly, only two surgically corrected intermittent exotropes exhibited a balanced pattern expected of normal controls (i.e., balance point > 0.9), while the remaining surgically treated patients exhibited different extents of sensory eye imbalance. Our results reveal that a functional eye imbalance still remains even in surgically corrected cases that have clinically normal stereopsis. Our study opens the possibility that a further treatment aimed at re-balancing the ocular dominance might be necessary for efficient binocular processing and binocular stability.

## Methods and Materials

### Participants

Fifty-three surgically aligned adults/teenagers with intermittent exotropia had been screened and ten of them (average age: 17.7 ± 5.3 years old; 4 females) who were successfully corrected and had normal clinical visual acuity and stereopsis were recruited to participate in the study. Normal stereo acuity was defined as less than 100 arc sec for clinical stereo tests using Random-dot stereograms (RDS test; Baoshijia, Zhengzhou, China) and Frisby (Baoshijia, Zhengzhou, China). They were measured at least three months after the surgery. A successful surgical alignment was defined as an exotropia of no more than 10 prism diopters at both far and near distance using the prism cover test. All cases had equal visual acuity in the two eyes, less than 2 diopters (D) of anisometropia, no previous history of strabismus surgery before the starting of the current study. Detailed characteristics are listed in Table 1. Another 10 normal adults (average age: 25.9 ± 2.5 years old; 6 females) with normal binocular vision and stereo acuity (40” in the Random-dot stereograms test) and no history of binocular dysfunction, participated in the study as controls. All treated patients and controls had normal or corrected normal visual acuity and fusion by Worth 4-Dot (Baoshijia, Zhengzhou, China).

All observers were naive to the purpose of the experiment. Written consent form was obtained prior to the study, which was approved by the Institutional Review Board of the Anhui Medical University in China, and the Institutional Review Board of the McGill University. The methods were carried out in accordance with the approved guidelines.

### Apparatus

All measurements were conducted on a PC computer running Matlab (MathWorks, Inc., Natick, MA) with PsychToolBox 3.0.9 extensions^34^,^35^. The stimuli were presented on a gamma-corrected LG D2342PY 3D LED screen (LG Life Science, Korea) with a 1920 × 1080 resolution and a 60 Hz refresh rate. Subjects viewed the display dichoptically with polarized glasses in a dimly lit room at a viewing distance of 136 cm. The background luminance was 46.2 cd/m^2^ on the screen and 18.8 cd/m^2^ through the polar glasses. A chin-forehead rest was used to minimize head movements during the experiment.

### Design

**Figure 1:**
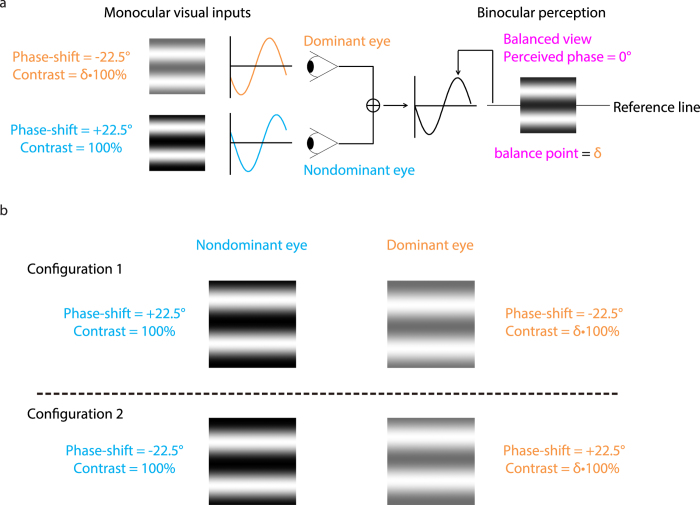
Illustration of the binocular phase combination paradigm. (**a**) Two horizontal sine-wave gratings with equal and opposite phase-shifts of 22.5° (relative to the center of the screen) were dichoptically presented to the two eyes through the polarized glasses. The perceived phase of the cyclopean grating depends on the internal representations of the two inputs. Sensory eye dominance is quantified by the interocular contrast difference that is needed to achieve a 0-degree of perceived phase, i.e., the balance point, where the two eyes are balanced in binocular combination. (**b**) To cancel any potential positional bias, two configurations were used in the measure: (1) the phase-shift was +22.5° in the nondominant eye and −22.5° in the dominant eye; (2) the phase-shift was −22.5° in the nondominant eye and +22.5° in the dominant eye. The perceived phase at each interocular contrast ratio (δ) was quantified by half of the difference between the measured perceived phases in these two configurations.

We quantitatively accessed the sensory eye dominance using a binocular phase combination paradigm^26^,^27^, which quantified the contributions of each eye to the fused binocular percept. The design was similar as the one we used in previous studies^30^,^32^, in which observers were asked to dichoptically view two horizontal sine-wave gratings having equal and opposite phase-shifts of 22.5° (relative to the center of the screen) through polarized glasses; the perceived phase of the grating in the cyclopean percept was measured as a function of the interocular contrast ratio. By this method, we were able to find a specific interocular contrast ratio where the perceived phase of the cyclopean grating was 0 degree indicating equal weight to each eye’s image. This specific interocular contrast ratio is the “balance point” for binocular phase combination since the two eyes under these stimulus conditions contribute equally to binocular vision (Fig. 1a). For each interocular contrast ratio, two configurations were used in the measurement so that any potential positional bias will be cancelled out (Fig. 1b): in one configuration, the phase-shift was +22.5° in the nondominant eye and −22.5° in the dominant eye and in the other, the reverse. The perceived phase of the cyclopean grating at each interocular contrast ratio (δ) was quantified by half of the difference between the measured perceived phases in these two configurations. Different conditions (configurations and interocular contrast ratios) were randomized in different trials, thus adaptation or expectation of the perceived phase would not have affected our results. The perceived phase and its standard error were calculated based on eight measurement repetitions.

Before the start of data collection, proper demonstrations of the task were provided by practice trials to ensure observers understood the task. During the test, observers were allowed to take short-term breaks whenever they felt tired.

### Stimuli

The gratings in the two eyes, as shown in Fig. 1, were defined as:


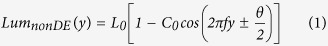



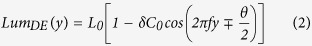


Where *L*_*0*_ is the background luminance; *C*_*0*_ is the base contrast in the nondominant eye; *f* is the spatial frequency of the gratings, *δ* is the interocular contrast ratio and *θ* is the interocular phase difference.

In our test, *L*_*0*_ = 46.2 cd/m^2^ (on the screen); *C*_*0*_ = 100%; *f* = 1 cycle/°; *δ* = [0, 0.1, 0.2, 0.4, 0.8, 1.0] and *θ* = 45°.

Surrounding the gratings, a high-contrast frame (width, 0.11°; length, 6°) with four white diagonal lines (width, 0.11°; length, 2.83°) was always presented during the test to help observers maintain fusion.

### Procedure

We used the same phase adjustment procedure as used by Huang *et al*.^27^ for measuring the perceived phase of the binocularly combined grating. In each trial, observers were asked firstly to align the stimuli from the two eyes; they were then instructed to adjust the position of a reference line to indicate the perceived phase of the binocularly combined grating. Since the gratings had a period of 2 cycles corresponding to 180 pixels, the phase adjustment had a step size of 4 degrees of phase/pixel (2 cycles × 360 phase-degree/cycle/180 pixels).

### Curve fits

The functions of perceived phase (*φ*) versus interocular contrast ratios (*δ*) i.e., the PvR functions, were fitted with a modified contrast-gain control model from Huang *et al*.^27^:


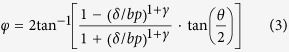


In which, *bp* and *γ* are two free parameters. ‘*bp*’ represents the interocular contrast ratio when the two eyes make equal contributions to binocular combination (i.e., the balance point) and ‘*γ*’ represents a non-linear factor.

Curve fitting was conducted in Matlab (MathWorks, Natick, MA) using the nonlinear least squares method to minimized ∑(*φ*_*theory*_ − *φ*_*observed*_)^2^. The goodness-of-fit was statistically tested by computing the *R-square* value:


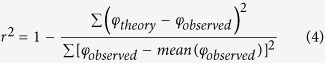


## Results

**Figure 2:**
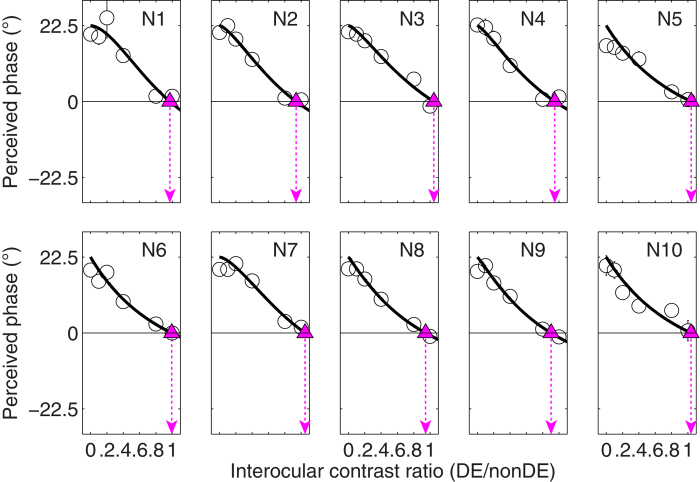
Binocular combination in normal adults. Binocular perceived phase of the cyclopean grating is plotted against interocular contrast ratio (dominant eye/nondominant eye) for 10 normal adults (N1-N10) in separate panels. The solid curve in each panel shows prediction from the contrast-gain control model. The purple triangle (▲) is the cross point of the horizontal black line and the solid curve. This indicates the effective contrast ratio at the balance point where the two eyes are equally effective. Error bars represent standard errors (some smaller than the data symbols).

The PvR functions for the 10 controls are plotted in Fig. 2. Consistent with previous studies in normal binocular adults using the same technique^26^,^27^,^28^,^29^,^30^,^31^,^32^,^36^, the perceived phase of the cyclopean grating gradually decreased from the phase of the nondominant eye (i.e., 22.5**°**) when there was no signal in the dominant eye (i.e., *δ* = 0) to be around zero when the two eyes had equal contrast (i.e., *δ* = 1). A repeated-measure within-subject ANOVA also revealed that the perceived phase depended strongly on the interocular contrast ratios (*F*(5,45) = 155.472, *p* < 0.001). The contrast-gain control model fits our data well with an average goodness-of-fit of 0.921 ± 0.058 (mean ± *SD*). Averaged across the 10 controls, the contrast ratio at the zero crossing point of the PvR curves (i.e., the balance point marked with purple arrows) is 0.987 ± 0.053 (mean ± *SD*). These results are consistent with previous measurements of interocular suppression in normal adults^26^,^27^,^28^,^29^,^30^,^31^,^32^,^36^, suggesting that the two eyes are almost functionally balanced.

**Figure 3:**
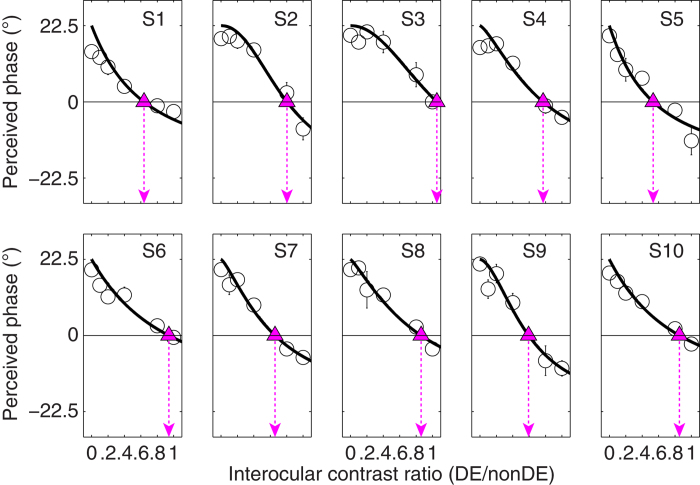
Binocular combination in surgically corrected intermittent intermittent exotropes. Binocular perceived phase is plotted against the interocular contrast ratio for 10 surgically corrected intermittent intermittent exotropes (S1–S10). Figure is organized in the same manner as Fig. 2.

**Figure 4:**
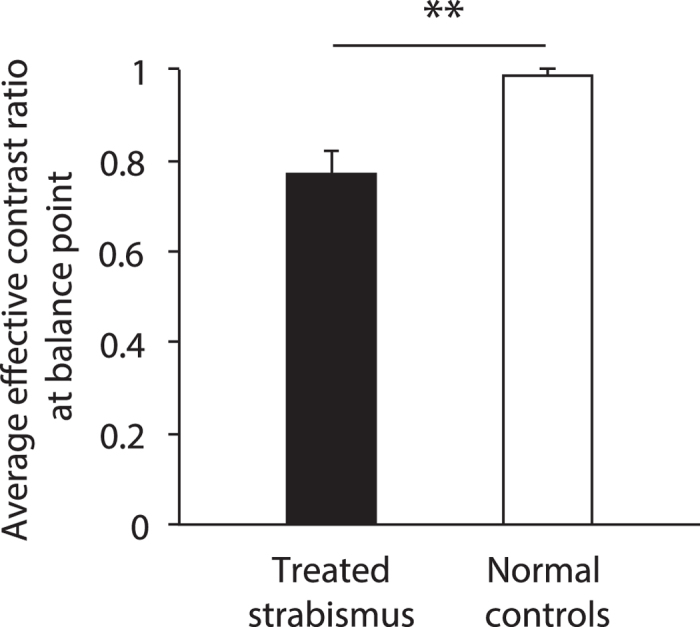
Different sensory eye dominance in surgically corrected intermittent exotropes and in normal adults. The two eyes of surgically corrected intermittent exotropes are significantly imbalanced compared with that of normal adults. “**” represents the result of a two-tailed t-test for two independent samples, *p* = 0.002.

The PvR functions for the 10 surgically corrected intermittent exotropes are plotted in Fig. 3. Similar contrast-dependency was also found in these treated patients in which the perceived phase decreased monotonically as the interocular contrast ratio increased (*F*(5,45) = 117.448, *p* < 0.001). However, except for observers S3 and S6, all others had a balance point that was shifted compared with the normal level (0.987). The contrast-gain control model also fits this patient data well with an average goodness-of-fit of 0.891 ± 0.075 (mean ± *SD*) and the average effective contrast ratio at balance point is 0.771 ± 0.164 (mean ± *SD*). A two-tailed t-test for two independent samples, corrected for inequality of variances based on Levene’s Test for Equality of Variances, revealed that the effective contrast ratio at the balance point was significantly different in the normal and patient group: t(10.873) = −3.959, *p* = 0.002 (Fig. 4). A between-subject repeated-measure ANOVA, with interocular contrast ratio as the within-subject factor and group as the between-subject factor, also showed that the PvR functions were significantly different between the two groups: F(1, 18) = 9.736, *p* = 0.006.

## Discussion

In this study, we quantitatively assessed the sensory eye dominance of surgically corrected intermittent exotropes who have normal stereo and visual acuity. Using a binocular phase combination paradigm, we show clear evidence that the two eyes are still unbalanced in most (8 out of 10) of the surgically corrected strabismics even though they have a normal range of stereo and visual acuity after the surgery. Such sensory eye imbalance is not likely to be due to inaccurate alignment of stimulus to the fovea of each eye, since an accurate left/right image alignment procedure always preceded the phase measurement in each trial. Therefore, the observed interocular imbalance in treated strabismics represents a residual sensory imbalance.

Some surgically treated patients had small tropias after surgery, yet they attained a random-dot stereopsis of 40 arc sec. This result agrees with a previous study from Pageau, de Guise and Saint-Amour^37^, which showed that about 70% of microtropes have normal local stereopsis to 20 sec of arc. This is either due to abnormal retinal correspondence during the tropia testing or because their tropias were resolved during the test. We did not monitor eye alignment during the test and so we cannot say for sure which of these two possibilities occurred.

Previous studies with this same paradigm have shown that the effective contrast ratio at balance point is far less than 1.0 (the ideal normal level) in amblyopes, for example, an average of 0.338 was found in a group of 11 adult amblyopes (with or without strabismus) by Zhou, Huang and Hess^32^ and the range was 0.11–0.28 for five anisometropic amblyopes in a study from Huang, Zhou *et al*.^27^. The magnitude of the binocular sensory imbalance in our treated non-amblyopic strabismics (i.e., 0.771 in average) was much less than previous reported in amblyopic strabismics, but still abnormal compared with the non-strabismic controls (our worst case was 0.903 in one of our normal controls), suggesting a subtler, sensory eye imbalance in this treated patient group.

The presence of an imbalance is a surprising finding in our treated strabismics all of whom have normal stereo acuity, as we would have thought an imbalanced ocular dominance would lead to reduced stereopsis^38^. Stereopsis is quite variable in the normal population with two well-defined peaks, one centered at 100 arc seconds and another at 700 arc seconds^39^. Defining normal stereopsis as 100 arc seconds and below is therefore justified even though a few individuals in the “normal” distribution are able to achieve stereo thresholds around 10–20 arc seconds. However, a more laboratory-based method of measuring stereopsis would be required to take these present findings to the next level, namely assessing what the relationship is between levels of stereopsis, all of which are within the normal range, to levels of ocular imbalance. What we can say is that in our study, using our clinical stereo measures, the treated patients and the normal controls were not significantly different in terms of their stereo performance across two independent tests: RDS (*p* = 0.138, 2-tailed independent samples t-test); Frisby (*p* = 0.081). However, they were significantly different in terms of their ocular balance (*p* = 0.002). Thus, our study provides additional insight into binocular function in this special patient population, namely, patients who previously had intermittent exotropia and were successfully surgically aligned, and now have normal visual acuity and stereo acuity. Our results suggest that even though they appear to be binocularly normal in terms of acuity, stereo and eye alignment, they possess a binocularly imbalanced input from each eye that is not previously recognized. Our data cannot answer whether the residual sensory imbalance was the same or not before surgery, i.e., whether there was an effect of surgery itself on sensory imbalance. This issue would need to be addressed in future work.

In conclusion, the sensory eye imbalance in treated strabismics with normal stereo and visual acuity is abnormal. This suggests that a small degree of suppression is present in patients who have an intermittent exotropia. Suppression of this magnitude does not in itself appear to lead to losses in visual acuity and its relationship to stereopsis is to be determined. It remains to be seen if this imbalance can be normalized by additional binocular treatment paradigms undertaken postsurgically.

## Additional Information

**How to cite this article**: Feng, L. *et al*. Sensory eye balance in surgically corrected intermittent exotropes with normal stereopsis. *Sci. Rep*. **5**, 13075; doi: 10.1038/srep13075 (2015).

## Figures and Tables

**Table 1:** Clinical details of the surgically corrected intermittent exotropes.

Subject#	Age/Sex	Cycloplegic Refractive Error (OD/OS)	Visual Acuity (OD/OS)	Preoperative deviation (OD/OS, prism dioper)		Postoperative deviation (OD/OS, prism dioper)		Postoperative Stereo Acuity (")	
				Near	Far	Near	Far	RDS - 40 cm	Frisby - 80 cm
S1	18/M	Plano	20/20	Ø	Ø	Ø	Ø	40	60
		Plano	20/20	XT50	XT50	XT5	XT8		
S2	24/M	−1.00DS	20/20	XT85	XT80	XT10	XT5	40	60
		−1.00DS	20/20	Ø	Ø	Ø	Ø		
S3	18/M	−1.00DS	20/20	XT50	XT55	Ø	XT3	40	40
		Plano	20/20	Ø	Ø	Ø	Ø		
S4	25/F	−2.50DS	20/20	Ø	Ø	Ø	Ø	60	40
		−1.50DS	20/20	XT50	XT50	XT2	XT4		
S5	13/F	−3.50DS	20/20	XT65	XT60	XT10	XT7	60	40
		−2.50DS	20/20	Ø	Ø	Ø	Ø		
S6	13/F	−3.50DS/−0.75DC×180	20/20	Ø	Ø	Ø	Ø	40	40
		−4.25DS/−1.25DC×180	20/20	XT60	XT55	XT2	XT4		
S7	18/M	−3.75DS	20/20	Ø	Ø	Ø	Ø	100	60
		−4.00DS	20/20	XT80	XT70	XT8	XT4		
S8	24/F	−0.50DS	20/17	Ø	Ø	Ø	Ø	40	40
		Plano	20/17	XT70	XT70	XT1	XT1		
S9	10/M	Plano	20/20	XT60	XT60	XT3	XT2	40	40
		Plano	20/20	Ø	Ø	Ø	Ø		
S10	14/M	−2.50DS	20/25	Ø	Ø	Ø	Ø	40	40
		−3.00DS	20/25	XT90	XT90	Ø	Ø		

Strabismus angle was measured using the prism cover test; All patients had intermittent exotropia (XT) before the surgery.
